# Thymine as potential biomarker to predict 5-FU systemic exposure in patients with gastro-intestinal cancer: a prospective pharmacokinetic study (FUUT-trial)

**DOI:** 10.1007/s00280-025-04759-8

**Published:** 2025-02-15

**Authors:** Maarten A. Hanrath, Evi Banken, Sebastian A. H. van den Wildenberg, Daan van de Kerkhof, Dirk Jan A. R. Moes, Michele Boisdron-Celle, Bianca J. C. van den Bosch, Ramon Bax, Pierre M. Bet, Jan Gerard Maring, Geert-Jan M. Creemers, Irene. E. G. van Hellemond, Maarten J. Deenen

**Affiliations:** 1 Department of Clinical Pharmacy, Catharina Hospital, Eindhoven, the Netherlands; 2 Department of Medical Oncology, Catharina Hospital, Eindhoven, the Netherlands; 3 Department of Clinical Chemistry, Catharina Hospital, Eindhoven, the Netherlands; 4 Laboratory of Chemical Biology, Department of Biomedical Engineering, Eindhoven University of Technology, Eindhoven, the Netherlands; 5 Department of Clinical Pharmacy and Toxicology, Leiden University Medical Center, Leiden, the Netherlands; 6 Department of Oncopharmacology-Pharmacogenetics INSERM U892, Western Cancer Institute Paul Papin, Angers, France; 7 Department of Clinical Genetics, Clinical Genomics, Maastricht University Medical Center (MUMC+), Maastricht, the Netherlands; 8 Department of Clinical Pharmacology and Pharmacy, Amsterdam University Medical Center, Amsterdam, the Netherlands; 9 Department of Clinical Pharmacy, Isala, Zwolle, the Netherlands; 10 GROW Research Institute for Oncology and Reproduction, Maastricht University, Maastricht, The Netherlands

**Keywords:** Gastrointestinal cancer, 5-Flurouracil, Endogenous DPD substrates, Toxicity, Thymine

## Abstract

**Purpose:**

In 20–30% of the patients, fluoropyrimidines (5-FU) based chemotherapy leads to severe toxicity, which is associated with dihydropyridine dehydrogenase (DPD) deficiency. Therefore, *DPYD* genotyping became standard practice before treatment with fluoropyrimidines. Nevertheless, only 17% of the patients with severe toxicity have a *DPYD* variant. Therefore, an urgent need persists to investigate other strategies contributing to prediction and prevention of toxicity. Endogenous DPD substrates are considered as potential biomarkers to predict toxicity, yet contradictional data exist on demonstrating uracil as a reliable biomarker. Thymine as biomarker for toxicity has been investigated less. The aim of this study was to determine the association between the concentrations of uracil, thymine dihydrouracil (DHU) and dihydrothymine (DHT), with the systemic drug exposure of 5-FU and DPD enzyme activity in patients treated with 5-FU.

**Methods:**

We included 36 patients with gastrointestinal malignancy who received 5-FU infusion. *DPYD* genotyping was conducted before start of treatment. Blood samples for determining 5-FU, uracil and thymine concentrations during infusion and DPD enzyme activity were taken.

**Results:**

We found a significant correlation between the 5-FU systematic exposure and baseline thymine concentrations (*R*^2^ = 0.1468; *p* = 0.0402). DPD enzyme activity was significantly correlated with baseline thymine concentrations but no correlation was found between DPD enzyme activity and 5-FU systemic drug exposure.

**Conclusion:**

5-FU dose individualization based on thymine concentrations could be a promising addition to *DPYD* genotyping to predict 5-FU-induced toxicity. Larger prospective trials are needed to examine thymine as predictor for toxicity in daily practice.

**Trial registration:**

Trial NL7539 at ‘Overview of Medical Research in the Netherlands’ (ID NL-OMON21471). Date of registration 19-02-2019.

## Introduction

The anticancer drug 5-fluorouracil (5-FU) and its oral prodrug capecitabine belong to the group of the fluoropyrimidines and are among the most commonly applied anticancer drugs. Up to 20–30% of colorectal cancer patients treated with 5-FU experience toxicity, such as mucositis, myelosuppression, and hand-foot syndrome. Moreover, in 0.05- 2% of the patients treated with 5-FU, toxicity even results in death [1–3]. Severe toxicity is strongly associated with deficiency of the primary 5-FU inactivating enzyme dihydropyrimidine dehydrogenase (DPD) [4–7]. The main known cause of DPD-deficiency is a genetic polymorphism within its encoding gene *DPYD* [8]. Pre-treatment genotyping of *DPYD* in combination with genotype-guided dosing has shown to significantly prevent severe and lethal toxicity of 5-FU-based chemotherapy [9, 10]. Based on these results, the European Medicines Agency has recently recommended that patients should be tested for DPD-deficiency before initiating treatment with 5-FU, and pre-treatment *DPYD* genotyping is standard of care and recently even mandatory in the Netherlands and most European countries [11, 12]. Nevertheless, a *DPYD* variant is detected in only 17% of the patients who experience severe toxicity, indicating that a large proportion of patients at risk for severe toxicity are not identified in advance [13]. This is due to the relatively low sensitivity of genotyping, as investigated by Meulendijks et al., who found a sensitivity of 6% for predicting severe toxicity, although specificity remains very high (95%) [14]. Another option to prevent patients from experiencing toxicity is to perform Therapy Drug Monitoring (TDM), as recently recommended by the International Association of Therapeutic Drug Monitoring and Clinical Toxicology [15]. An area under the curve (AUC) below the therapeutic 5-FU exposure range has been associated with worse treatment outcome, while an AUC above this range has been associated with a higher risk for severe toxicity. However, TDM for 5-FU is not described in guidelines, and is not routinely conducted. In addition, it is only applicable for intravenous fluoropyrimidines administration, and not for oral fluoropyrimidines use, including capecitabine and S-1. Therefore, there remains an urgent need to further enhance personalized fluoropyrimidines based treatment in order to prevent severe toxicity.

Besides genotyping, also phenotyping approaches exist including assessment of the DPD enzyme activity. Studies have shown that variations in DPD enzyme activity correlate to 5-FU plasma concentrations [7, 16, 17]. In turn, increased 5-FU plasma concentrations are associated with increased 5-FU-induced toxicity [18–20]. Therefore, measurement of DPD enzyme activity in peripheral blood mononuclear cells (PBMCs) may also be a valuable tool for assessment of DPD-deficiency. However, measurement of the DPD enzyme activity in PBMCs is a rather costly and time-consuming assay compared to genotyping. In addition, DPD enzyme activity measurements require specific resources, making it unfortunately less suitable for use in daily clinical practice [13].

**Fig. 1:**
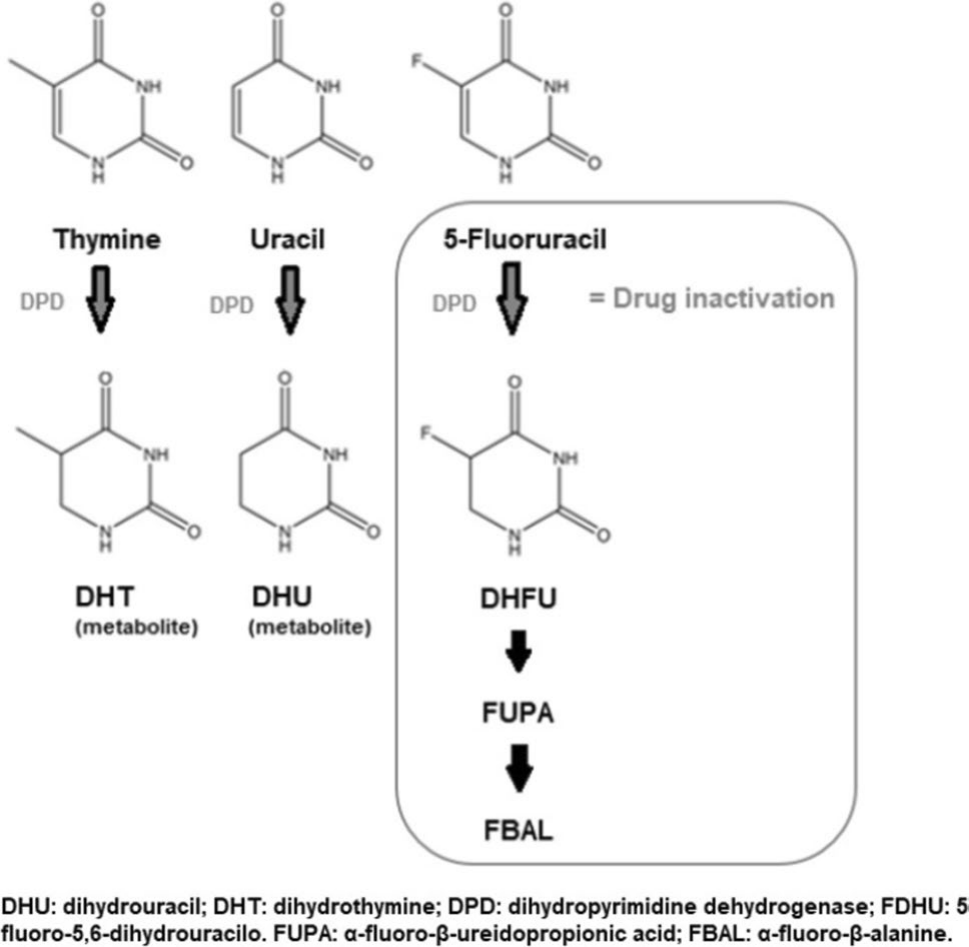
Metabolism of uracil, thymine and 5-fluorouracil effected by DPD-enzyme

Another phenotyping approach to identify DPD-deficiency is by measuring the concentrations of endogenous DPD substrates, i.e. uracil and thymine in blood plasma. Like 5-FU, these endogenous DPD substrates are converted by the DPD enzyme (Fig. 1) [21]. Several studies have investigated the association between uracil and/or its ratio with its metabolite dihydrouracil (DHU) and 5-FU induced toxicity [14, 22–24]. Although the definitive role of uracil as a biomarker is not well-defined yet, it is currently subject of investigation to apply an initial dose reduction in patients with uracil pre-treatment concentrations > 16 ng/mL [14, 25]. However, the positive and predictive value of pre-treatment uracil concentrations appear lower compared to genotyping, and studies show contradictory results [13, 25, 26]. These contradictory findings are partly explained by variations in pre-analytical sample handling, which is a critical factor affecting results of endogenous uracil concentration measurements [27]. A falsely increased concentration of uracil may result in less optimal 5-FU treatment [25, 28]. Besides, also other factors such as renal impairment, food intake, liver resection, and circadian rhythm all significantly influence uracil concentrations [29–31]. Based on all existing data, the clinical utility of pre-treatment uracil has not yet been demonstrated. Most previous studies focused on uracil and DHU concentrations, whereas almost no data exist that support the expected similar association between the concentration of DPD substrate thymine and DPD-deficiency or 5-FU-induced toxicity.

In order to further determine whether the endogenous DPD substrate thymine may be a more consistent and predictive biomarker for DPD-deficiency, this study aimed to determine the correlation between the baseline endogenous DPD substrate plasma (ratio) concentrations of thymine, uracil, DHU and dihydrothymine (DHT) and the systemic drug exposure of 5-FU and DPD enzyme activity in patients with gastrointestinal cancer treated with intravenous 5-FU-based chemotherapy.

## Material and methods

### Study design

This was a prospective, single-center, non-randomized pharmacokinetic study. The primary goal of this study was to establish the correlation between the baseline endogenous DPD substrate plasma (ratio) concentrations of uracil, thymine and their metabolites DHU and DHT, with the systemic drug exposure of 5-FU as defined by the area under the plasma concentration time-curve (AUC) in patients with pancreas or colorectal cancer treated with intravenous 5-FU-based chemotherapy. Secondary objectives were the correlation between DPD enzyme activity with the uracil, thymine, and 5-FU concentrations, and to describe the change in concentrations of the biomarkers over time during 5-FU infusion. In addition, toxicity of the treatment was assessed clinically. All patients provided written informed consent. The study involving human participants was conducted in accordance with the Declaration of Helsinki, and was approved by the Ethics Committee of the Medical Research Ethics Committees United Nieuwegein (Registration number A19.017/R19.002). The protocol was also approved by the local ethics committee. The study was registered at the Netherlands Trial Register (https://trialsearch.who.int/Main ID: NL7539).

### Patient population

Patients were eligible to participate if they had a gastrointestinal malignancy for which they were intended for treatment with 2-weekly cycles of 5-FU continuous infusion in the FOLFOX, FOLFIRI or FOLFIRINOX treatment regimen. Inclusion criteria were age 18 years or older, WHO performance status of 0–2, and minimum laboratory values at baseline: absolute neutrophil count ≥ 1.5 × 10^9^/L, platelet count ≥ 100 × 10^9^/L, hepatic function as defined by serum bilirubin ≤ 1.5 × ULN, ALAT and ASAT ≤ 2.5 × ULN, in case of liver metastases ALAT and ASAT ≤ 5 × ULN, and renal function (CKD-EPI) > 30 mL/min. Patients were excluded when the bolus 5-FU administration was omitted, had known history of substance abuse or psychotic disorders that would prevent follow up, or had other disease or conditions that could negatively interfere with the study or the patient’s safety.

### Treatment procedure

All patients received standard of care treatment according to local treatment protocols based on international treatment guidelines. The 5-FU dose regimen in all treatment schedules was 400 mg/m^2^ as a 15-min bolus injection followed by 2400 mg/m^2^ as continuous infusion for 44 h. The complete treatment schedules are shown in Table 4 (appendix). Patient’s demographic data, medical history, hematology and chemistry laboratory assessments were performed prior to start of 5-FU treatment, hematology and chemistry laboratory tests were measured at each cycle. Before start of treatment with 5-FU, all patients were genotyped for DPD-deficiency by analysis of the four single nucleotide polymorphisms (SNPs) most strongly associated with DPD-deficiency, i.e. DPYD*2A, DPYD*13, 2846A > T and 1236G > A (HapB3 haplotype). Based on the patients’ genotype the *DPYD* gene activity score (between 0–2) was calculated, and patients with a reduced gene activity score were treated with an initially reduced 5-FU starting dose as described by Henricks et al. [32].

### Measurement of 5-FU pharmacokinetics and the endogenous biomarkers.

EDTA blood samples for determining 5-FU, its metabolite dihydrofluorouracil (DHFU) and the biomarkers uracil, thymine, DHU and DHT plasma concentrations were obtained at baseline, and at time *t* = 0.5, 2 and 44 h after start of the continuous 5-FU infusion. Immediately after blood collection, samples were stored on ice and then centrifuged for 10 min at 2000 *g*. The obtained plasma was stored at −80 °C until analysis. Samples were analyzed using a validated LC–MS/MS method described by Remaud et al. and van den Wildenberg et al., respectively [21, 33]. Pharmacokinetic parameters were estimated using a previously described 2-compartment population pharmacokinetic model based on the 5-FU and DHFU concentration–time data, respectively by non-linear mixed-effects modeling using NONMEM (V7.4.4, Icon Development Solutions, Ellicot City, MD, USA) [34]. The Perl-speaks-NONMEM toolkit version 4.8.1 and Pirana version 2.9.7 were used as modeling environment. Results were plotted using R statistics (v4.2.1, Boston, MA, USA) and RStudio (2024.04.2 build 764). Exposure (AUC0-44) was calculated using the individual predicted values (concentration) and time data using trapezoidal rule with R statistics.

### Measurement of DPD enzyme activity

EDTA blood samples for determining DPD enzyme activity were taken at baseline. The DPD enzyme activity samples were analyzed within 48 hours after blood drawl according to the method described by Coenen et al., with a mean ± SD enzyme activity of 15.2 ± 5.7 nmol/mg protein/hour and a reference range for normal of 8.70–24.40 nmol/mg protein/hour [35].

### Toxicity evaluation

5-FU-related toxicity was assessed every treatment cycle for the first 3 treatment cycles by analysis of the individual patient health care records. Toxicity, including thrombocytes, leukocytes, neutrophils, diarrhea, nausea and mucositis, was assessed according by the Common Terminology Criteria for Adverse Events of the National Cancer Institute (NCI-CTC-AE v4.03) [36]. For association analyses with DPD enzyme activity, pharmacokinetic (PK) and endogenous biomarkers, toxicity outcomes were categorized into hematological toxicity and gastrointestinal toxicity and analyzed as in no or mild toxicity (grade 0–2) versus severe toxicity (grade ≥ 3). Considering that reduced dosing in *DPYD* variant carriers results in similar systemic drug exposure as wild type patients treated with a full dose, calculated AUC’s in *DPYD* variant carriers were not dose-corrected for the association analysis with toxicity [9].

### Statistical analysis

All data collected were recorded in the electronic case report form (research manager version 5.53.0.7). Correlation tests were determined using the Pearson correlation coefficient with a two-tailed (95% confidence interval). An unpaired T-test was used to compare DPD enzyme activity and toxicity. For the correlation test between endogenous DPD substrates and their ratio’s with systemic 5-FU exposure, the 5-FU AUCs in *DPYD* variant allele carriers treated with reduced doses were dose-corrected to allow proper comparison. Correlation tests were analyzed using the Wilcoxon test and *P*-values < 0.05 were considered statistically significant. All data was analyzed by using GraphPad Prism version 10.0.2 software (GraphPad Software LLC, San Diego, CA, USA).

## Results

A total of 48 patients were assessed for eligibility in the study of which 7 patients were excluded due to meeting an exclusion criterion. Four patients did not receive 5-FU bolus therapy, in 2 patient blood collection failed and 1 patient only received 1 cycle of chemotherapy. A total of 41 patients entered the study, in which PK sampling and 5-FU concentration measurements were evaluable for the study in 36 patients. There were two patients with a *DPYD* mutation (2846A > T), who received a 5-FU dose intensity of 75%.

### Patient characteristics

Of all 36 patients, 23 were male (63.9%) and 13 were female (36.1%). The median age was 62 years (range 41–79). All patients were Caucasian. The primary tumor type in most patients was colorectal cancer (22 patients) followed by pancreatic cancer (8 patients). All baseline characteristics are summarized in Table 1.

**Table 1:** Baseline patient characteristics

	*n* = 36	%
Gender		
Male	23	63.9
Female	13	36.1
Age (mean ± SD in years)	62 ± 7.3	
Ethnicity		
Caucasian	36	100
Primary tumor type		
Colorectal	22	61.1
Pancreatic	8	22.2
Other^a^	6	16.7
Current stage of cancer		
Local	6	16.7
Locally advanced	6	16.7
Metastatic	24	66.7
Treatment schedule		
FOLFOX	14	38.9
FOLFIRI	15	41.7
FOLFIRINOX	7	19.4
Targeted therapy		
None	28	77.8
Bevacizumab	5	13.9
Trastuzumab	1	2.8
Panitumumab	2	5.6
*DPYD* genotype		
Wild type	34	94.4
Heterozygous (2846A > T)	2	5.6

^a^Other primary tumor types: esophageal cancer, gastric cancer

*FOLFIRI* 5-FU, irinotecan, *FOLFIRINOX* 5-FU, oxaliplatin, irinotecan, *FOLFOX* 5-FU, oxaliplatin, *SD* standard deviation

### Correlations between endogenous DPD substrate concentrations and 5-FU systemic drug exposure

**Fig. 2:**
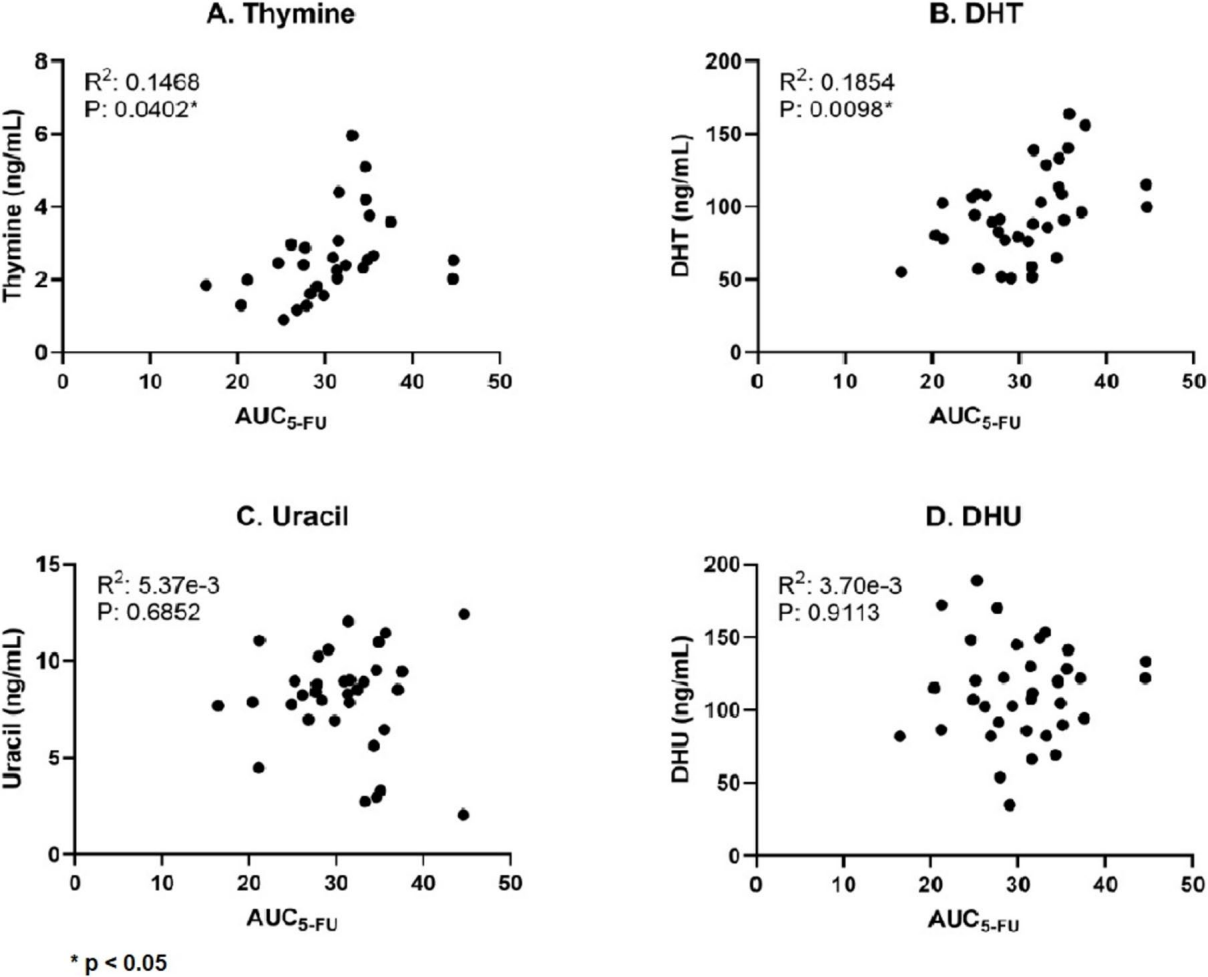
The correlations between the concentrations of the biomarkers versus AUC of 5-FU

The baseline plasma concentrations of the endogenous DPD substrate concentrations and the 5-FU systemic drug exposure are shown in Table 2. Uracil and thymine were both normally distributed. There was a significant correlation between the 5-FU systemic exposure (AUC) and baseline thymine plasma concentration (*R*^2^ = 0.1468; *p* = 0.0402), as well as with the baseline DHT concentration (*R*^2^ = 0.1854; *p* = 0.0098), shown in Fig. 2. The uracil, DHU nor the DHT/T and DHU/U ratios correlated significantly with systemic drug exposure.

**Table 2:** Concentrations of the endogenous biomarkers and 5-FU systemic drug exposure

	*n*	Concentration
Baseline concentration endogenous biomarkers (mean ± SD in µg/L)		
Uracil	34^a^	8.4 ± 3.4
DHU	36	112.7 ± 33.6
Thymine	30^a^	2.8 ± 1.7
DHT	35^a^	95.0 ± 29.3
DHU/U	34	38.67 ± 14.15
DHT/T	30	16.22 ± 10.57
AUC_0–44_ (mg/L × h)		
5-FU	36	29.8 ± 5.2
DHFU	36	48.8 ± 8.9
Pharmacokinetic parameters (median (IQR))		
Distribution Volume (V_d_) (L)	36	52.5 (51.5–53.4)
Clearance (Cl) (L/h)	36	30.7 (24.7–43.8)
Q (L/h)	36	111.9 (102.0–139.5)
DPD-enzyme activity (median (IQR) in nmol/mg protein/hour)	36	14.2 ± 4.4 (6.2 – 22.4)

^a^Measurements of endogenous biomarkers did not succeed in all patients

*AUC* Area under the curve, *5-FU* 5-fluorouracil, *DHFU* dihydrofluorouracil, *DHU* Dihydrouracil, *DHT* dihydrothymine, *Q* intercompartmental clearance

### Correlation between endogenous DPD substrate concentrations and DPD enzyme activity

DPD enzyme activity was normally distributed with an average DPD enzyme activity of 14.2 ± 4.4 nmol/mg protein/hour and range of 6.2–22.4 nmol/mg protein/hour. Baseline thymine concentration is correlated significantly with DPD enzyme activity (*R*^2^ = 0.195; *p* = 0.016). The other endogenous DPD substrate concentrations nor the ratios correlated significantly with DPD enzyme activity (Table 3).

**Table 3:** Correlations of DPD enzyme activity with the baseline endogenous biomarkers concentrations and with the 5-FU AUC_0–44_

	Pearson *r*	*R*^2^	*P* (two tailed)
Biomarker			
Uracil	− 0.0007	0.000	0.997
DHU:U	0.1826	0.033	0.309
Thymine	− 0.4421	0.195	0.016
DHT:T	0.3219	0.104	0.095
5-FU AUC_−0–44_	0.0603	0.0036	0.727

*DHU* Dihydrouracil, *DHU:U* ratio DHU/uracil, *DHT* dihydrothymine, *DHT:T* ratio DHT/thymine, *AUC* area under the curve

### Correlation between DPD enzyme activity and 5-FU exposure

The DPD enzyme activity and AUC of 5-FU did not correlate significantly, as presented in Table 4 and Appendix Fig. 5.

### Endogenous DPD substrate concentrations during 5-FU infusion

**Fig. 3:**
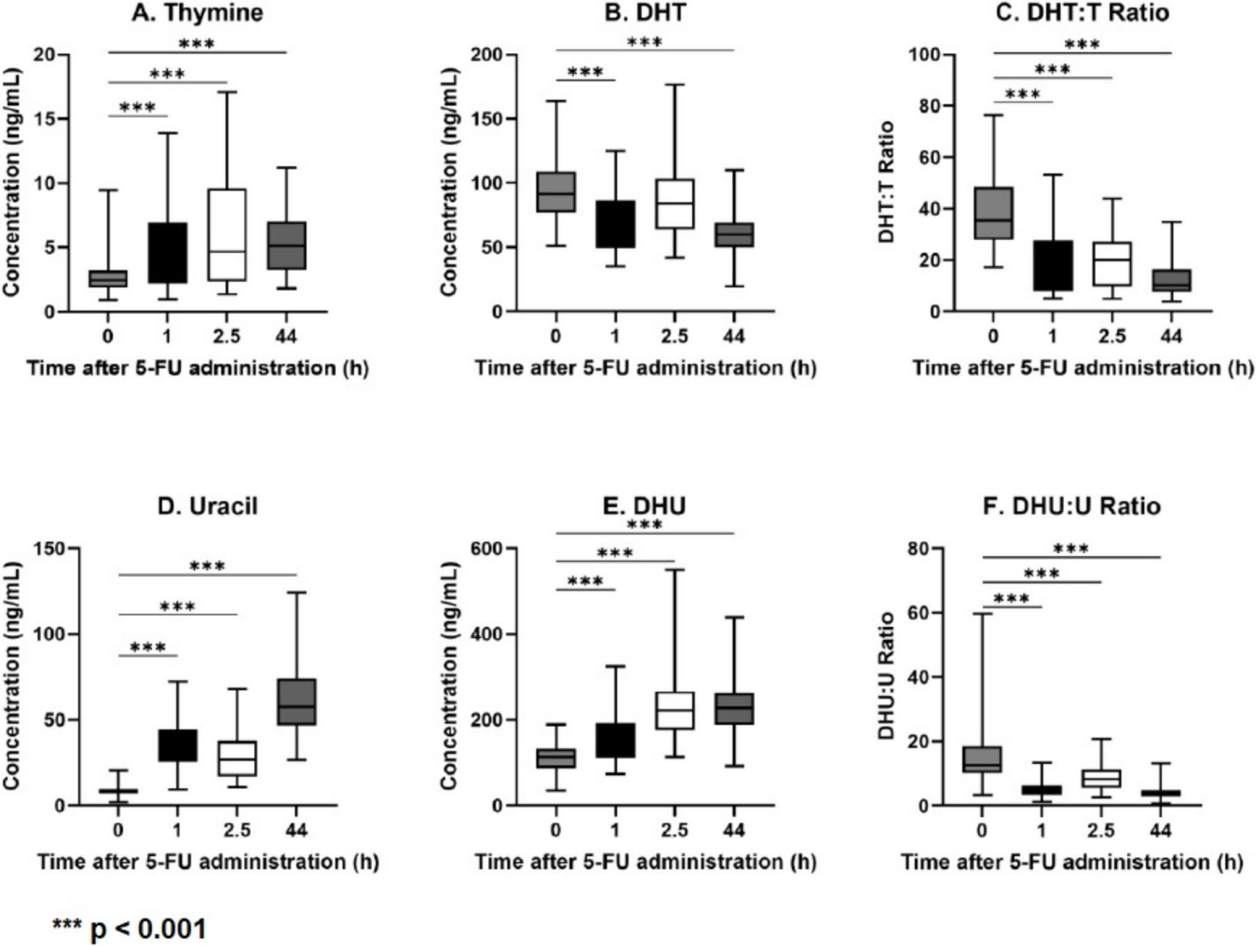
The concentration of the biomarkers plotted against time

The concentrations of the endogenous biomarkers during 5-FU infusion are shown in Fig. 3. The thymine concentrations increased over time compared to baseline, while its metabolite DHT significantly decreased during 5-FU infusion. As a result of the above finding, the ratio DHT/T was significantly lower during 5-FU infusion as compared to baseline (*p* < 0.001). Also, uracil showed an increase in concentration over time, and in contrast to DHT, DHU concentrations increased during 5-FU infusion. Also here, the ratio DHU/U was significantly lower during 5-FU infusion as compared to baseline.

### Toxicity evaluation

A total of twelve (33%) patients experienced severe toxicity (grade ≥ 3) and 24 (67%) patients experienced mild toxicity (grade 1–2). Mild (grade 1–2) and severe (grade ≥ 3) gastrointestinal toxicity occurred in 21 (58%) and ten (28%) patients, respectively; mild and severe hematological toxicity occurred in 31 (86%) and 4 (11%) patients, respectively. Eleven of the 36 patients (31%) required a 5-FU dose reduction in subsequent treatment cycles, of which four patients (11%) were admitted to the hospital due to toxicity. The hospitalization duration varied between 4 and 10 days. All individual toxicity outcomes are described in Table 5 (appendix). Severe gastrointestinal and/or hematological toxicity was plotted against baseline thymine concentrations, uracil concentrations and the AUC^0−44^ of 5-FU (Appendix Fig. 4). No visual correlation was observed between severe toxicity and baseline thymine concentrations or the AUC of 5-FU. In contrast, a visual correlation between severe toxicity and baseline uracil concentrations was observed.

## Discussion

This study analyzed the correlations between DPD endogenous substrates and the systemic drug exposure of 5-FU in patients with gastrointestinal malignancies. Using endogenous biomarkers as strategy for dose-individualization is a matter of interest since *DPYD* genotyping does not identify all DPD-deficient patients [13, 37]. With this study we made a first step in prospectively investigating the endogenous biomarker thymine as a potential predictor for 5-FU exposure and its related toxicity. Our results show there is a significant correlation between the endogenous DPD substrate concentrations thymine and 5-FU systemic drug exposure. The fact that thymine, similar to 5-FU, is metabolized by the enzyme DPD, suggests that thymine can be a potential biomarker to predict 5-FU-induced toxicity.

Compared to the current literature, previous studies mainly focused on using uracil as biomarker to detect DPD-deficiency rather than thymine. Two earlier studies found that uracil plasma concentrations and the DHU/U ratio can effectively identify patients with reduced DPD metabolism [20, 38]. Etienne-Grimaldi and Meulendijks et al. also concluded plasma uracil concentrations can function as predictor for severe grade IV toxicity in patients treated with 5-FU [14, 26]. However, recently, the Alpe2U study evaluated a 50% dose reduction of fluoropyrimidine in patients with high pretreatment uracil concentrations [24]. Although they found a low incidence of severe toxicity in patients with a normal *DPYD* genotype and uracil levels > 16 ng/mL, it was associated with a 56% lower AUC of 5-FU. They concluded dose individualization based on uracil levels may result in underdosing and measuring endogenous uracil concentrations requires specific equipment which is not widely available in hospitals. In addition, Wildenberg et al. observed that ex-vivo uracil concentrations continue to rise over time as the conversion of uridine to uracil by uridine phosphorylase remains active until the plasma is chemically processed or stored in the freezer [21]. Despite this, the study of Kuilenburg et al. still demonstrated a significant correlation between uracil and DPD enzyme activity and did not find a correlation with thymine concentrations [39]. The post-sampling metabolic activity and instability of uracil in blood and plasma, may partly explain the variability in study results regarding correlations with uracil and 5-FU exposure. Uracil is also more sensitive to pre-analytical variation compared to thymine concentration measurements [21, 31, 40, 41]. Additionally, recently it was found that both thymine and uracil are partially bound to proteins as was determined by protein ultrafiltration, where recovery differences can be related to the extent of molecule protein binding [42]. It can therefore be suggested that uracil provides important challenges that need to be properly addressed for use in daily clinical practice.

Given the known challenges of using uracil as reliable biomarker for indicating DPD-deficiency, our study focused on studying thymine as biomarker to predict 5-FU exposure. We found a significant correlation between thymine and 5-FU exposure, suggesting thymine can function as potential biomarker for prediction of severe toxicity. This hypothesis is supported by Wildenberg et al., who observed that thymine and DHT are more stable than uracil and DHU, and may offer a higher diagnostic accuracy to identify DPD-deficiency [21]. A previous study investigating thymine as biomarker used a thymine challenge test wherein patients received a 250 mg oral dose of thymine. Subsequently, thymine plasma and urinary concentrations were measured and compared with endogenous U/DHU levels and *DPYD* genotype [43, 44]. In line with our results, they found that the thymine challenge test is more sensitive in identifying severe toxicity and may serve as a better biomarker for 5-FU exposure than *DPYD* genotyping and endogenous-based uracil biomarkers. A comparable alternative over endogenous uracil concentrations is the uracil challenge test using an uracil loading dose, which also showed to be effective in identifying patients with reduced DPD activity [45]. Nevertheless, importantly, one should only use baseline biomarker concentrations for proper assessment, since our results demonstrated that thymine and uracil concentrations increased during 5-FU infusion. This can be explained by the fact that thymine and uracil both compete with 5-FU for conversion by DPD. This was also demonstrated by Thomas et al., who noted an increase of uracil concentrations after a median delay of 35 days between measuring the baseline sample and the sample during treatment. Nonetheless, in our study, variation in uracil and thymine concentrations already occurred shortly after start of 5-FU infusion, and underscores the observation that only baseline samples are suitable for diagnostic purposes [46]. We found an unexpected positive correlation between DHT and 5-FU exposure, which may be explained by intra-individual variation in the further metabolism of DHT by dihydropyrimidinase (DHP) [21].

Although DPD enzyme activity significantly correlated with baseline thymine concentrations, we could not demonstrate a correlation between DPD enzyme activity and the AUC of 5-FU. This may partly be explained by the relatively large variability in measured DPD enzyme activity as observed in this study (i.e. 6.2–22.4 nmol/mg protein/hour). The mean ± SD value of the assay is reported to be 15.2 ± 5.7 nmol/mg protein/hour [35]. It shows that DPD enzyme activity measurements are accompanied with a relatively large variability in measured activity, which may hamper to demonstrate clear correlations with e.g. systematic 5-FU drug exposure. Several factors may explain this variation, which consists of both analytical as well as biological variation. It is known that analytical accuracy firstly depends on the percentage of lymphocytes in the lysate; a lower percentage of lymphocytes results in lower measured DPD enzyme activity [47]; thus far, no correction factor can be applied for lower percentages of lymphocytes. Second, storage time after blood drawl also affects measured enzyme activity; notwithstanding, all patient samples were analyzed within maximally 48 h after blood drawl. Besides, in this analytical variation also biological variation exists, including circadian rhythm of DPD [29]. Moreover, no formal standardized reference value exists yet for DPD enzyme activity. For example, two other DPD enzyme activity assays described in literature show an average measured DPD enzyme activity of 9.9 ± 2.8 nmol/mg protein/hour [47] or 9.6 ± 2.2 nmol/mg [48]. These differences highlight that uniformity in reference values, as well as precision and accuracy of the method used for DPD enzyme activity measurements, are important for proper use in clinical practice.

In this study, we made an attempt to investigate the relationship between the presence of severe toxicity and thymine concentrations at baseline. We hypothesized that high thymine concentrations would be associated with increased risk of severe toxicity (≥ grade 3). However, probably due to a small sample size and heterogenous treatment regimens consisting of both doublet as well as triplet treatment regimens, we were not able to demonstrate a clear relationship between high thymine concentrations and toxicity. Although we did observe a visual correlation between baseline uracil concentrations and severe toxicity, the study was not powered to assess associations with toxicity, and thus no statistical tests were conducted. Yet, the observed association for uracil aligns with previous data on this biomarker. Based on the correlation between thymine and 5-FU concentrations we found in this study, we are still convinced of the hypothesis that high thymine concentrations might be associated with severe toxicity. Hence, larger prospective trials are needed to confirm this hypothesis. Despite pretreatment *DPYD* genotype-guided dosing as part of routine care in this study, we still observed significant severe toxicity incidences as is known from 5-FU treatment. This further underscores that the current *DPYD* genotyping guidelines should be expended with additional (rare) *DPYD* variants to increase sensitivity of DPD testing [49]. This again emphasizes the need for further research regarding strategies for predicting 5-FU induced toxicity. Another limitation of this study might be the limited sampling strategy applied for determination of 5-FU systemic exposure. However, by using NOMNEM models we managed to determine an accurate systemic exposure of 5-FU.

In conclusion, *DPYD* genotyping took a first step towards better prediction of severe 5-FU-induced toxicity. Nevertheless, a significant proportion of the patients experiencing severe toxicity are not carriers of a *DPYD* variant and therefore adequate dosing is still challenging. Our results provide additional insights into dose-individualization strategies based on thymine concentrations, which may complement on *DPYD* genotyping in efforts to improve the safety of 5-FU-based chemotherapy. While the measurement of thymine concentrations appears to be more consistent and accessible compared to other endogenous DPD substrates such as uracil, further research is needed to validate its clinical utility. At this stage, combining *DPYD* genotyping with thymine measurements is a promising concept, but its effectiveness in preventing 5-FU-related toxicity remains to be confirmed. Larger prospective trials are needed to examine the sensitivity of thymine as a predictive biomarker for 5-FU-induced toxicity and to develop implementation in daily practice, in order to achieve a more tailored therapy approach.

## Figures and Tables

**Fig. 4:**
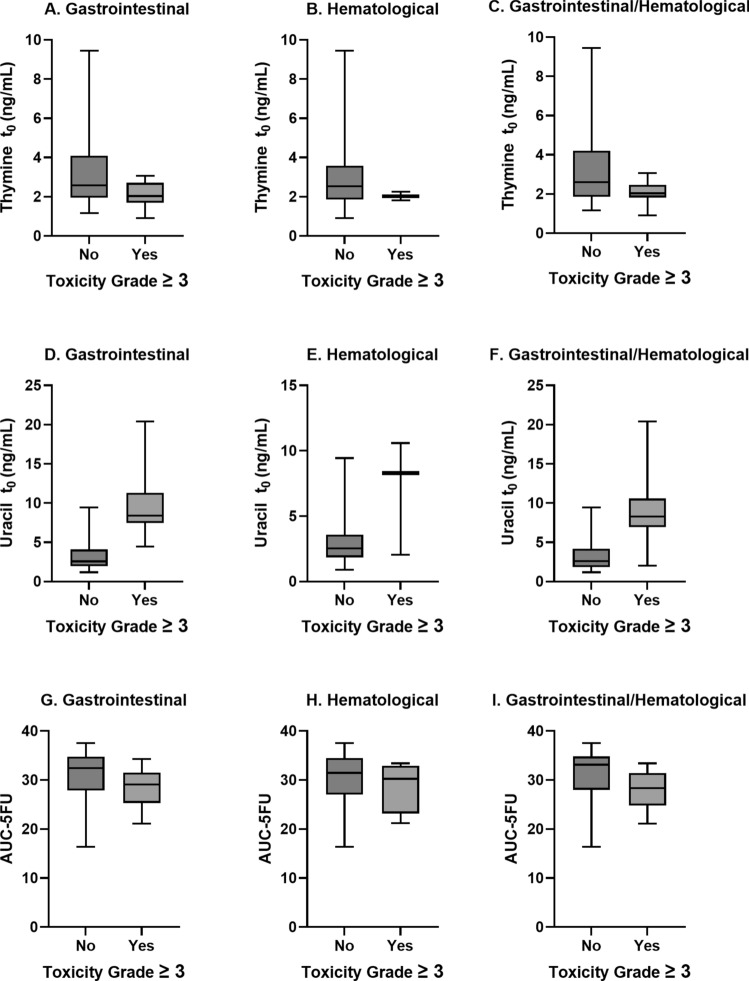
Correlations between thymine concentrations and 5-FU AUC with 5-FU related severe toxicity

**Fig. 5:**
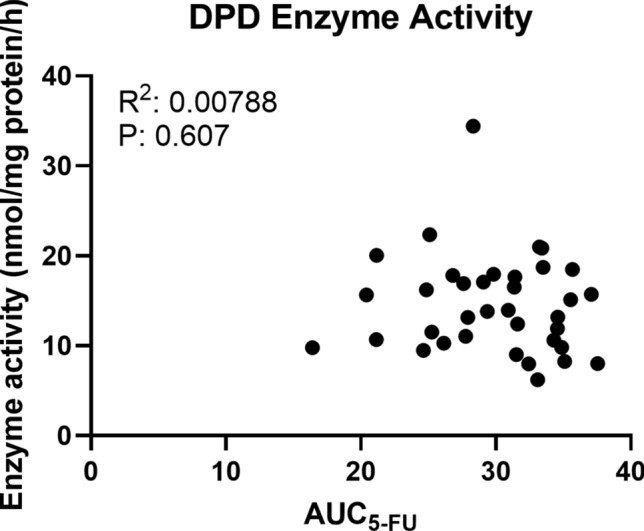
Correlation between DPD enzyme activity and AUC of 5-FU

## Data Availability

Data that support the findings of this study are available from the corresponding author upon reasonable request.

## Appendix

See Tables 4, 5 and Figs. 4, 5.

**Table 4:** Dosing schedules FOLFOX, FOLFIRI and FOLFIRINOX conform local protocol

Regimen	Folinic acid	5-FU bolus	5-FU infusion	Oxaliplatin	Irinotecan	Other^a^
	mg/m^2^	mg/m^2^	mg/m^2^	mg/m^2^	mg/m^2^	mg/kg
FOLFOX 6 modified	400	400	2400	85	–	–
FOLFOX 6 bevacizumab	400	400	2400	85	–	5
FOLFOX panitumumab	400	400	2400	85	–	6
FOLFIRI	400	400	2400	–	180	
FOLFIRI bevacizumab	400	400	2400	–	180	5
FOLFIRI panitumumab	400	400	2400	–	180	6
FOLFIRI ramucirumab	400	400	2400	–	180	8
FOLFIRINOX	400	400	2400	85	180	–
FOLFIRINOX modified	400	400	2400	85	150	–

^a^B = bevacizumab; P = panitumumab; R = ramucirumab; all are dosed in mg/kg body weight

**Table 5:** 5-FU related toxicity within the first 3 cycles of treatment

5-FU related toxicity	Grade	*n* (36)	%
Diarrhea Supplementary	0 1–2 ≥ 3	13 16 7	36.1 44.4 19.4
Mucositis	0 1–2 ≥ 3	25 9 2	69.4 25.0 5.6
Nausea/vomiting	0 1–2 ≥ 3	20 14 2	55.6 38.9 5.6
Myelosuppression	0 1–2 ≥ 3	34 2 –	94.4 5.6 –
Anemia	0 1–2 ≥ 3	32 4 –	88.9 11.1 –
Thrombocytopenia	0 1–2 ≥ 3	35 1 –	97.2 2.8 –
Leukopenia	0 1–2 ≥ 3	33 2 1	91.7 5.6 2.8
Neutropenia	0 1–2 ≥ 3	31 3 2	86.1 8.3 5.6
Other^a^	0 1–2 ≥ 3	15 18 3	41.7 50.0 8.3

^a^Fatigue, cardiac chest pain, dysgeusia, epistaxis

## Abbreviations

5-FU: 5-Fluorouracil
AUC: Area under the curve
DHFU: Dihydrofluorouracil
DHT: Dihydrothymine
DHT/T: Dihydrothymine/thymine
DHU: Dihydrouracil
DHU/U: Dihydrouracil/uracil
DHP: Dihydropyrimidinase
DPD: Dihydropyrimidine dehydrogenase
DPYD: Dihydropyrimidine dehydrogenase gene
FOLFIRI: 5-FU, irinotecan
FOLFIRINOX: 5-FU, oxaliplatin, irinotecan
FOLFOX: 5-FU, oxaliplatin
PK: Pharmacokinetic
